# Sex Differences in the Association Between Childhood Cyberbullying Victimization and College Sexual Assault: Moderating Roles of Parental Monitoring and Relationship with Deviant Peers

**DOI:** 10.1177/10778012261418689

**Published:** 2026-03-03

**Authors:** Jungup Lee, Debra Patterson, Na Youn Lee, Jinyung Kim, Stephen J. Tripodi, Hyunkag Cho

**Affiliations:** 1 Department of Social Work, 37580 National University of Singapore, BLK AS3 Level 4, 3 Arts Link, Singapore 117569, Singapore; 2 Social Service Research Centre, 37580 National University of Singapore, BLK AS7 Level 3, 5 Arts Link, Singapore 117570, Singapore; 3 School of Social Work, 2954 Wayne State University, 5447 Woodward Avenue, Detroit, MI 48202, USA; 4 School of Social Work, 7161 San Jose State University, One Washington Square, Rm 215, San Jose, CA 95192, USA; 5 Anne and Henry Zarrow School of Social Work, 6187 University of Oklahoma, 700 Elm Ave. Zarrow Hall, Norman, OK 73069, USA; 6 College of Social Work, 7823 Florida State University, 296 Champions Way, University Center Building C2500, Tallahassee, FL 32306, USA; 7 School of Social Work, 3078 Michigan State University, 655 Auditorium Road, 230 Baker Hall, East Lansing, MI 48824, USA

**Keywords:** childhood cyberbullying victimization, college sexual assault, sex difference, parental monitoring, deviant peer association

## Abstract

This study explores sex differences in the relationship between childhood cyberbullying victimization (CCBV) and young adult sexual assault experiences and the potential moderating roles of childhood parent monitoring and deviant peer association on the relationship. A total of 356 college students aged 19–25 in the US participated in the online survey. The results indicated that CCBV was associated with an increased risk of college sexual assault victimization for both males and females. A significant moderating effect of childhood parental monitoring and deviant peer association was found between the association between CCBV and college sexual assault victimization for female students only.

## Introduction

Cyberbullying is an increasing concern for young people that extends beyond school walls. As such, more studies are starting to recognize both traditional bullying victimization and cyberbullying victimization as part of adverse childhood experiences [ACEs] (Stopbullying.gov, 2017). While there are many similarities between traditional bullying and cyberbullying, there are some significant variations between the two. Anonymity is a key feature that sets these categories apart (Kowalski et al., 2019). Most victims of cyberbullying do not know the dominant offender in bullying situations; thus, it is more difficult to solve certain cyberbullying cases. Another important distinction is that cyberbullying can occur during any time of the day without respite, whereas traditional bullying is mainly limited to school hours (Kowalski et al., 2019). Since cyberbullying follows the victims wherever they are, it could be more detrimental to individual victims (Hong et al., 2016; Lee, 2021). While many previous literature indicated that cyberbullying is significantly associated with negative mental and behavioral health outcomes (Cénat et al., 2021; Hébert et al., 2016; DePaolis & Williford, 2019; Elgar et al., 2014; Lee, 2021; Lee, Kim & Lee, 2025), positive parent or peer associations were also found to be buffering factors moderating this particular relationship (Lee et al., 2018; Wright, 2016).

### Definition and Prevalence of Cyberbullying

Cyberbullying is often defined as “an aggressive, intentional act toward a group or individual, using electronic forms of contact, repeatedly and overtime against a victim who cannot easily defend himself or herself” (Smith et al., 2008, p. 376). Meanwhile, cyberbullying victimization refers to being the target of aggressive or harmful behavior carried out by a group or individual through any form of electronic communications devices (e.g., receiving mean messages, being socially excluded by someone using social media platforms, becoming the subject of unpleasant gossip or rumors) (Lee et al., 2017). Due to the rising use of information and communication technologies (ICT) in recent society and unique characteristics of cyberbullying such as anonymity, young people are at greater risk of cyberbullying victimization than in the past (Lee, 2021; Lee, Choo, Zhang, et al., 2025). Furthermore, the global transition to online learning platforms and virtual social groups since the outbreak of the COVID-19 pandemic has redefined the significance and omnipresence of virtual spaces for school-aged children.

Although the prevalence of traditional bullying victimization is still higher than cyberbullying victimization (Eyuboglu et al., 2021; Jadambaa et al., 2019; e.g., 7.02% and 17% for cyberbullying victimization and 25.1% and 33% for traditional bullying victimization), the prevalence of cyberbullying victimization has been continuously rising due to the key features of cyberbullying and rampant usage of digital technologies over the past two decades. For instance, Hong et al. (2016) found that nearly 10% of students in grades 6–10 experienced cyberbullying victimization at least once. According to the 2017 National Youth Risk Behavior Survey (YRBS), 14.9% of high school students reported being cyberbullied through either texting, Instagram, Facebook, or other social media during the past year (Kann et al., 2018). In particular, there was a significantly higher prevalence of cyberbullying victimization experiences among female students (19.7%) compared to among male students (9.9%) (Kann et al., 2018). Similarly, Lee et al. (2021) found that there was a higher proportion of females (10.7%), compared to that of males (7.4%), who were victims of cyberbullying.

### Outcomes Associated with Cyberbullying

The rising trend of cyberbullying among young people is of serious concern because cyberbullying victimization causes various negative consequences that are comparable to traditional bullying. Victims of cyberbullying show high levels of depression, general anxiety, and other psychological distress (Cénat et al., 2021; Hébert et al., 2016). For example, a study of 660 3^rd^ graders and 960 5^th^ graders found that cyberbullying victimization resulted in significant negative effects on the children's school connectedness and health-related outcomes, such as low self-esteem and depression (DePaolis & Williford, 2019). Similarly, a meta-analysis of 27 longitudinal studies conducted by Lee, Choo, Zhang, et al. (2025) revealed significant longitudinal effects of cyberbullying victimization on multiple mental health and behavioral health problems among young people, including anxiety, depression, loneliness, psychological distress, somatic complaints, sleep problems, and stress. Various research has also shown that childhood traumatic experiences are known to cause serious behavioral and mental health issues among victims later in life (Lee, Munisamy, Ai, et al., 2025; Takizawa et al., 2014). As indicated, past investigations found that victims of cyberbullying were more likely to report mental health problems (Elgar et al., 2014; Lee, 2021). Specifically, childhood cyberbullying victimization is now considered a critical issue that leads to the following long-lasting adverse consequences: high levels of depression, anxiety, loneliness, suicide and sexual harassment, and the jeopardization of wellbeing over time (DePaolis & Williford, 2019; Kowalski et al., 2019; Lee, Munisamy, Ai, et al., 2025). Although there has been some evidence that supports the association between childhood bullying and sexual victimization (Moore et al., 2017), there is a dearth research on the influence of childhood cyberbullying victimization on later sexual assault as a college student. Overall, childhood bullying is a traumatic experience that may lead to severe consequences that can significantly impact the victims’ future. Thus, the purpose of this study is to examine the association between childhood cyberbullying victimization and college sexual assault experiences, and what factors moderate this association.

### Relationship Between Cybervictimization and Sexual Assault as College Students

Previous studies have consistently reported that college students are at a high risk of being sexually assaulted (Fedina et al., 2018; Franklin et al., 2012; Hines et al., 2012). According to the 2019 Association of American Universities (AAU) Climate Survey on Sexual Assault and Sexual Misconduct, a national survey of 181,752 students across 33 universities, 13% of all students, including undergraduate, graduate, and professional respondents, reported having experienced sexual violence in college, with higher rates reported by females and sexual minorities (Cantor et al., 2019). Female undergraduates were three times more likely to be subject to nonconsensual sex by force or inability to consent than female graduate or professional students (25.9% vs 9.7%); whereas 22.8% and 14.5% of undergraduate and graduate/professional students, respectively, who identify as sexual minorities reported having been subject to such victimization (Cantor et al., 2019). These two groups were also more likely to experience sexual harassment (59.2% for female undergraduates vs. 65.1% for sexual minorities); intimate partner violence (IPV) (14.1% vs. 21.5%); and stalking (10% vs. 15.2%).

Examining the relationship between childhood cybervictimization together with sexual assault is important in that childhood sexual and emotional victimization is tied to adult sexual assault (Niehaus et al., 2010). Although research has not examined the relationship between childhood cyberbullying victimization and college sexual assault, given that cyber victimization is significantly associated with number of dating partners and number of sexual partners (Lapierre & Dane, 2020), there could be a tentative association between cyber victimization and sexual assault. Additionally, the ACE pyramid developed by Centers for Disease Control and Prevention [CDC] postulates that ACEs influence health and well-being throughout the lifespan, including social/emotional/cognitive impairment, adoption of health risk behavior, disease, disability, social problems, and early death (CDC, 2021). Connecting this framework to cybervictimization and sexual assault, it is possible to infer that being the victim of sexual assault later as an adult could be the outcome of health risk behavior or social problem. Further, the statistics showed that 11.2% of all college students experience sexual assault through physical force, violence, or incapacitation (Cantor et al., 2019). Given the high rate of sexual violence in the college years and the paucity of research on the relationship between cyberbullying victimization and college sexual assault, it is imperative to explore the link between childhood cyberbullying and sexual assault in emerging adulthood to inform prevention efforts.

Out of many factors associated with young adults’ sexual assault victimization, including living on campus, frequently drinking until drunk, being unmarried, and having been sexually assaulted prior to the school year (Cantor et al., 2019; Hines et al., 2012), one of the most consistent predictors of adult sexual revictimization is childhood victimization (Messman-Moore & Brown, 2004). Female college students with a history of childhood sexual or emotional victimization were more likely to report adult rape than their counterparts who were not victimized in childhood (Messman-Moore & Brown, 2004). Niehaus et al. (2010) explain that this could be due to the negative self-image childhood sexual abuse (CSA) survivors have of their sexuality. Compared to nonvictims, CSA survivors tended to view themselves as sexually immoral, irresponsible, dirty, less romantic/passionate, open, and less conservative (Niehaus et al., 2010). However, most studies have focused on the impacts of childhood victimization on adult sexual revictimization for females, and there is a dearth of studies on sex differences in the association between childhood victimization and college sexual assault. Although the prevalence of sexual assault for male students was lower than that for female students, nearly one in 15 male undergraduates were victims of sexual violence in college (Cantor et al., 2019). Existing studies also reveal that male college students are sexually victimized, often by female perpetrators or same-sex perpetrators (Hines et al., 2012; Weiss, 2010). Thus, a more comprehensive investigation on sex differences in college sexual assault experiences should be investigated.

### Moderating Role of Parent and Peer Influences

Although the moderating roles of parental monitoring and relationship with deviant peers were not explored in many studies, it is possible to infer their moderation effects based on a bulk of literatures that support the strong association between parental monitoring with cybervictimization, and deviant peers with cybervictimization. Because parents and peers have a strong influence on the wellbeing of youth (Elsaesser et al., 2017; Lee, 2021; Rudolph et al., 2014), studies have examined their role on the effects of cyberbullying. Parent influences can buffer against youth's cyberbullying victimization and sexual crime (Jankowski et al., 2002; Kowalski et al., 2019). Positive parent-child relationships and parental monitoring are protective variables to mitigate the risk of cyberbullying victimization (Kowalski et al., 2014). In addition, parental support plays a vital role in reducing victimization, even when peer support is negligible. For example, a longitudinal study of 1,416 adolescents examined the interaction between peers’ social support and parental social support and found that children who received family social support had decreased cyberbullying victimization even when they had low levels of peer social support (Fanti et al., 2012). As it relates to peers, peer associations have been linked to bullying victimization. The term “*homophily*” explains the inclination of children and adolescents who tend to prefer friends that are like them (Veenstra & Dijkstra, 2011). For instance, according to a U.S. longitudinal study (Rudolph et al., 2014), bullying victimization was associated with an increased level of deviant peer association by a homophily selection process. Also, another study with 1,401 adolescents showed that bullying victimization had a positive association with deviant peer association (Zhu et al., 2016).

Parent and peer factors, such as parental monitoring and peer deviance, may mitigate or deteriorate the association between bullying and negative outcomes. Positive parent or peer associations can alleviate the link between cyberbullying and risk behaviors, while negative associations can enhance this link. For instance, Wright (2016) found that both a higher level of parental support and a higher number of close friends moderated the association between cyberbullying victimization and substance use. Furthermore, parental monitoring and deviant peer association were found to be significant moderators on the link between cyberbullying and substance use in a recent study (Lee et al., 2018). Lee et al. (2020) indicated that peer support was found to buffer the relationship between cyberbullying victimization and school dropout intention. Although parent and peer influences have been identified as crucial moderators to the impact of cyberbullying victimization on substance use and school dropout, there has been a dearth of research which examined the moderating effect of these variables on the association between cyberbullying victimization and sexual assault.

### The Current Study

Empirical research on cyberbullying in youth has increased over the past decade, but little is known about the behavioral patterns and changes in subsequent sexual violence in emerging adulthood. The current study aims to examine and understand sex differences in the relationship between childhood cyberbullying victimization (CCBV) and sexual assault experiences among college students. Drawing upon prior research, the moderating effects of childhood parental monitoring and deviant peer association on the relationship are also examined. We derive the following hypotheses. First, we hypothesize that females will have more CCBV and sexual assault experiences in college than males. Second, we hypothesize that CCBV will be positively associated with college sexual assault experiences for both males and females, after controlling for both sociodemographic and online behavior variables. Third, we hypothesize that childhood parental monitoring will be negatively associated with college sexual assault experiences for both males and females, whereas childhood deviant peer association will be positively associated with college sexual assault experiences for both males and females. Lastly, we hypothesize that childhood parental monitoring will buffer the relationship between CCBV and sexual assaults, while childhood relationship with deviant peers will exacerbate the association between CCBV and sexual assault in emerging adulthood.

## Method

### Sample and Procedure

The sample for this study included college students aged 19–25 from two large public universities in a southeastern U.S. state. To be more specific, a random sample of 2000 university students from two large southeastern universities (1000 each) in the U.S. was selected and emailed an invitation to participate in a web-based survey. College students were eligible to participate in the study if they were junior or senior undergraduate students enrolled in one of the two large public universities, were between the ages of 19 to 25, and used digital technology in everyday life. The sample population excluded freshmen and sophomores because this is a transition time period from late adolescence to young adulthood and thus, freshmen and sophomores may not accurately capture their cybervictimization experiences during childhood (Lee, 2021).

The current study used the retrospective and cross-sectional online survey method to inquire about the participants’ CCBV and sexual assault experiences during their college career. To recruit participants using random sampling, we contacted the universities registrars’ offices and submitted the required forms. Afterward, we attached a detailed description of the study, an online survey, and the informed consent forms to be dispersed via emails in the blind carbon copy. The online survey was then created using Qualtrics. The participants took approximately 30 minutes to complete their surveys. The study was approved by the university institutional review board.

The final sample for this study consisted of 356 randomly sampled undergraduate students. As indicated in Table 1, the sample consisted of 69.7% female participants, 56.5% non-Hispanic Whites, and 11.5% sexual minorities including gay/lesbian, bisexual, and prefer not to specify. Approximately 36% of the sample were low-income undergraduate students who received or were eligible for income-based grants for their tuition or living expenses (e.g., a Pell Grant, Federal Supplemental Education Opportunity Grant). The mean age of the sample was 20.7 years (*SD* = 1.29).

**Table 1.** Descriptive Statistics.

	Total (N = 356)	Male (n = 108)	Female (n = 248)	χ^2^ or t-test
	n (%) or M ± SD	n (%) or M ± SD	n (%) or M ± SD	
Age (19–25)	20.66 ± 1.29	20.89 ± 1.48	20.56 ± 1.19	2.07*
Sex				
Male	108 (30.3)			
Female	248 (69.7)			
Race/ethnicity				11.60**
White (non-Hispanic)	201 (56.5)	59 (54.6)	142 (57.3)	
Black (non-Hispanic)	30 (8.4)	2 (1.9)	28 (11.3)	
Latinx	63 (17.7)	22 (20.4)	41 (16.5)	
Others	62 (17.4)	25 (23.1)	37 (14.9)	
Sexual orientation				5.13
Heterosexual/straight	309 (88.5)	90 (86.5)	219 (89.4)	
Gay/lesbian	8 (2.3)	5 (4.8)	3 (1.2)	
Bisexual	25 (7.2)	6 (5.8)	19 (7.8)	
Prefer not to specify	7 (2.0)	3 (2.9)	4 (1.6)	
Income-based grant				0.94
Yes	124 (35.6)	37 (35.9)	87 (35.5)	
No	224 (64.4)	66 (64.1)	158 (64.5)	
Hours spent online (1–24)	7.57 ± 3.73	7.90 ± 3.97	7.41 ± 3.62	1.07
Perceived online safety (0–6)	3.99 ± 1.58	4.53 ± 1.50	3.76 ± 1.54	4.31***
Childhood parental monitoring (1–5)	4.46 ± 0.76	4.42 ± 0.75	4.47 ± 0.77	-0.53
Childhood deviant peer association (1–5)	1.59 ± 0.47	1.73 ± 0.56	1.53 ± 0.41	3.55***

### Measures

#### Childhood Cyberbullying Victimization

Childhood cyberbullying victimization (CCBV) was measured with the 9-item Cyberbullying Victimization (CV) scale developed by Hinduja and Patchin (2009). This scale enquired to rate the respondents’ experiences of being victimized through internet use in childhood (i.e., before the age of 18). Some example statements include “received an instant message that made me upset” and “had something posted on social media platforms such as Facebook that made me upset”. Each item was rated on a 5-point Likert scale ranging from 1 = never, 2 = once or twice, 3 = a few times, 4 = many times, and 5 = every day. All responses were first recoded dichotomously to “non-involved” or “involved” based on the cut-off point of “a few times”. Then, responses were summed and used as count data ranging from 0 to 9 (α = .88 for total respondents, α = .89 for male participants, and α = .88 for female participants).

#### College Sexual Assault Experiences

College sexual assault experiences were measured with a modified 8-item version of the Sexual Experience Survey (SES; Koss et al., 1987). This scale revolved around the respondents’ experiences of being sexually assaulted in their college years (e.g., “verbally coerced sexual intercourse”, “forced sexual contact”, and “being raped”). There were six response categories for each item: 0 = never, 1 = 1 time, 2 = 2 times, 3 = 3 times, 4 = 4 times, and 5 = 5 times or more. All responses were recoded dichotomously to “yes” (i.e., have been sexually assaulted in college years) or “no” (i.e., have not been sexually assaulted in college years) and were composited as count data, which ranged from 0 to 8 (α = .86 for total respondents, α = .82 for male participants, and α = .86 for female participants).

#### Childhood Parental Monitoring

Childhood parental monitoring was measured with the 6-item Parental Monitoring Scale (PMS; Li, Stanton & Feigelman, 2000). This scale inquired about the respondents’ perceptions of parents’ tracking and supervision of their whereabouts and activities during childhood (e.g., “My parent (s) knew where I was after school” and “I told my parent (s) who I was going to be with before I went out”). Each item was rated on a 5-point Likert scale which ranges from 1 = never to 5 = always. A composite indicator of childhood parental monitoring was shaped by averaging across items, with a higher score indicating a higher level of childhood parental monitoring (α = .93 for total respondents, α = .92 for male participants, and α = .93 for female participants).

#### Childhood Association with Delinquent Peers

Childhood association with delinquent peers was measured with the 12-item Antisocial Behavior subscale of the 19-item Peer Delinquent Behavior (PDB) scale developed for the Rochester Youth Development Study (Thornberry et al., 1994). This scale indicated the extent to which the respondents associated with peers who engaged in antisocial activities during childhood (e.g., “hit someone with the idea of hurting them”, “damaged or destroyed someone else's property on purpose”, and “got drunk”). The items were rated on a 5-point scale ranging from 1 = none of them to 5 = all of them, and scores were averaged to indicate levels of childhood deviant peer association (α = .86 for total respondents, α = .89 for male participants, and α = .82 for female participants).

#### Covariates

Covariates for this study included sociodemographic and online behavior variables. The five sociodemographic variables were *age* (in years); *sex* (male/female); *race/ethnicity* (non-Hispanic White, non-Hispanic Black, Latinx, Others); *sexual orientation* (heterosexual/straight, gay/lesbian, bisexual, prefer not to specify); and *expected family contribution* (EFC; “Are you currently a recipient or eligible for a Pell Grant, Federal Supplemental Education Opportunity Grant, or other income-based grant?”; yes/no). The two variables that measured online use were *hours spent online* (2 items; “How many hours a day do you usually use the Internet on a weekday” and “How many hours a day do you usually use the Internet and on a weekend”; an averaged composite score ranging from 0 to 24 hours a day) and *perceived online safety* (6 items; e.g., “Do you feel safe when you make friends through Facebook or Instagram?”; yes/no; a compound score ranging from 0–6; α = .73 for total respondents, α = .74 for male participants, and α = .70 for female participants).

### Data Analyses

Data analyses were performed using STATA software version 16.0. The maximum likelihood (ML) estimation with robust standard errors was used to treat missing values. Descriptive analyses were performed to ensure the prevalence of all the primary variables. The Waldo chi-square test and *t*-test analyses were conducted to compare the estimates of major variables between male participants and female participants. Zero-inflated Poisson regression analyses were utilized to examine the association between CCBV and college sexual assault experience. The zero-inflated Poisson model was available in the case of a larger probability of zero scores for count data (Hernandez-Avila et al., 2006). In the Poisson models, incidence rate ratios (IRRs) were calculated to determine the ratio of the frequency in which an event occurs in the proposed population at any given time (Lee et al., 2018). In Model 1, CCBV and covariates were entered together. In Model 2, we examined the roles of childhood parental monitoring and deviant peer association in the incident rates of sexual assault experiences. In Model 3, the two-way interaction terms between CCBV, the parent factor, and the peer factor were added to investigate the moderating effects of childhood parent and peer influences on the association between CCBV and sexual assault experiences. All analyses were conducted separately by sex.

## Results

### Descriptive Statistics

Table 2 presents the prevalence of CCBV and college sexual assault experiences by sex. The prevalence rates of CCBV indicated that female students (39.1%) reported slightly higher CCBV than male participants (38%); yet, there was no significant difference between male participants and female participants. Nearly 20.4% of male participants and 20.2% of female participants reported one or two incidents of CCBV out of nine incidents. Female participants (23.4%) reported the higher prevalence of the item, “having something posted on social network sites about them” compared to male participants (16.7%; χ^2^ = 3.02, *p* < .05), while male participants (17.6%) had the higher rate of the item, “being made fun of in a chat room” compared to female participants (8.1%; χ^2^ = 7.00, *p* < .01).

**Table 2.** Prevalence of Childhood Cyberbullying Victimization and College Sexual Assault Experiences.

Childhood cyberbullying victimization	Total n (%)	Male n (%)	Female n (%)	χ^2^	College sexual assault experiences	Total n (%)	Male n (%)	Female n (%)	χ^2^
1. I received an upsetting email from someone I know.	25 (7.0)	6 (5.6)	19 (7.7)	1.11	1. Having sex play without your consent because of threatening or physical force from your partner.	30 (8.6)	6 (5.8)	24 (9.8)	1.48
2. I received an instant message that made me upset.	94 (26.4)	29 (26.9)	65 (26.2)	0.16	2. Having intercourse without your consent because your partner was so sexually aroused.	84 (24.3)	16 (15.5)	68 (28.0)	6.10**
3. I had something posted on SNS (e.g., Facebook or Twitter) that made me upset.	76 (21.3)	18 (16.7)	58 (23.4)	3.02*	3. Being given in to intercourse when you did not want to because you were overwhelmed by your partner's continual arguments and pressure.	70 (20.2)	9 (8.7)	61 (25.0)	11.89***
4. I was made fun of in a chat room.	39 (11.0)	19 (17.6)	20 (8.1)	7.00**	4. Having your partner attempt sexual intercourse with you when you did not want to by threatening or using physical force.	20 (5.6)	3 (2.9)	17 (7.0)	2.19
5. I received an upsetting email from someone I did not know (not spam).	22 (6.2)	6 (5.6)	16 (5.6)	1.04	5. Having sexual intercourse when you did not want to because your partner threatened or used physical force.	14 (4.0)	-	14 (5.7)	6.16**
6. I had something posted about me on another web page that made me upset.	33 (9.3)	11 (10.2)	22 (8.9)	1.55	6. Engaging in sex acts (anal or oral sex) when you did not want to because your partner threatened or used physical force.	21 (6.1)	1 (1.0)	20 (8.2)	6.69**
7. Something had been posted about me online that I did not want others to see.	48 (13.5)	14 (13.0)	34 (13.7)	1.36	7. Having sexual intercourse when you were unable to give your consent, because you were drunk or taking drugs at the time, or because you are a heavy sleeper, or because you were unconscious for any reason.	65 (18.8)	13 (12.6)	52 (21.4)	3.65*
8. I was picked on or bullied online.	56 (15.7)	19 (17.6)	37 (14.9)	1.41	8. Being raped.	23 (6.6)	5 (4.9)	18 (7.4)	0.75
9. I was afraid to go on the computer due to cyberbullying experiences.	17 (4.8)	5 (4.6)	12 (4.8)	0.10					
Sum of all 9 items				*t*	Sum of all 8 items				*t*
None	218 (61.2)	67 (62.0)	151 (60.9)	8.48	None	223 (64.6)	82 (79.6)	141 (58.3)	18.84**
1	49 (13.8)	16 (14.8)	33 (13.3)		1	44 (12.8)	8 (7.8)	36 (14.9)	
2	23 (6.5)	6 (5.6)	17 (6.9)		2	26 (7.5)	5 (4.9)	21 (8.7)	
3	18 (5.1)	4 (3.7)	14 (5.6)		3	25 (7.2)	3 (2.9)	22 (9.1)	
4	16 (4.5)	2 (1.9)	14 (5.6)		4	7 (2.0)	1 (1.0)	6 (2.5)	
5	13 (3.7)	6 (5.6)	7 (2.8)		5	6 (1.7)	3 (2.8)	3 (1.2)	
6	10 (2.8)	4 (3.7)	6 (2.4)		6	3 (0.9)	-	3 (1.2)	
7	3 (0.8)	-	3 (1.2)		7	8 (2.3)	1 (1.0)	7 (2.9)	
8	3 (0.8)	2 (1.9)	1 (0.4)		8	3 (0.9)	-	3 (1.2)	
9	3 (0.8)	1 (0.9)	2 (0.8)						

The results revealed that there were significant differences by sex in the prevalence rates of college sexual assault experiences (see Table 2). More specifically, female students had more sexual assault experiences than male students did (*t* = 18.84, *p* < .01). Of the sample, 20.4% of male participants and 41.7% of female participants had experienced college sexual assaults. Most respondents who experienced college sexual assaults reported one to three incidents (15.6% of males; 32.7% of females). Interestingly, 1.2% of female participants experienced all the eight incidents of college sexual assault experiences. Among the eight items, 15.5% of male participants and 28% of female participants had sexual intercourse without their consent because their partner was so sexually aroused (χ^2^ = 6.10, *p* < .01), and 12.6% of male participants and 21.4% of female participants had sexual intercourse when they were unable to give their consent due to drunkenness, drug use, or any other reasons (χ^2^ = 3.65, *p* < .05).

### Sexual Assault Experiences in Zero-Inflated Poisson Regression Models

Tables 3 and 4 display the zero-inflated Poisson regression analyses investigating college sexual assault experiences associated with CCBV, covariates, and childhood parent and peer factors. The moderating effects of childhood parental monitoring and deviant peer association are also depicted.

**Table 3.** Zero-Inflated Poisson Regression Analyses Predicting College Sexual Assault Experiences.

	Total sample		
	Model 1	Model 2	Model 3
	IRR (95% CI)	IRR (95% CI)	IRR (95% CI)
CCBV	2.53 (2.19, 2.92)***	2.20 (1.88, 2.57)***	1.73 (1.35, 2.23)***
Age	1.02 (1.01, 1.20)*	1.10 (1.01, 1.20)*	1.10 (1.01, 1.20)*
Female	2.24 (1.65, 3.05)***	2.29 (1.67, 3.12)***	2.26 (1.64, 3.11)***
Race/ethnicity			
Black (non-Hispanic)	0.61 (0.38, 0.98)*	0.84 (0.52, 1.37)	0.88 (0.54, 1.44)
Latinx	0.56 (0.40, 0.78)**	0.51 (0.36, 0.72)***	0.52 (0.37, 0.74)***
Other	0.62 (0.43, 0.91)*	0.59 (0.41, 0.87)**	0.59 (0.40, 0.85)**
Sexual orientation			
Gay/lesbian	1.13 (0.61, 2.10)	0.90 (0.48, 1.69)	0.87 (0.46, 1.62)
Bisexual	1.38 (0.96, 1.98)	1.29 (0.89, 1.86)	1.23 (0.84, 1.79)
Prefer not to specify	1.13 (0.57, 2.24)	1.39 (0.69, 2.80)	1.40 (0.70, 2.81)
Income-based grant	1.44 (1.14, 1.82)**	1.30 (1.03, 1.65)*	1.27 (0.99, 1.61)
Hours spent online	1.03 (1.01, 1.06)*	1.03 (1.01, 1.06)*	1.03 (1.01, 1.06)*
Online safety	0.96 (0.90, 1.03)	0.96 (0.89, 1.03)	0.96 (0.89, 1.03)
Parental monitoring		0.62 (0.48, 0.79)***	0.55 ().38, 0.81)**
Deviant peer association		1.59 (1.21, 2.09)**	1.15 (0.77, 1.72)
CCBV × parental monitoring			0.55 (0.31, 0.96)*
CCBV × deviant peer association			1.85 (1.12, 3.06)*
χ^2^ (*df*)	213.57 (12)***	250.54 (14)***	259.48 (16)***
Pseudo *R^2^*	0.19	0.22	0.23

*Note*. IRR = incidence rate ratio; CI = confidence interval; CCBV = childhood cyberbullying victimization. Reference groups for sex, race/ethnicity, and sexual identity are male, White, and heterosexual/straight, respectively.

*p < .05. **p < .01. ***p < .001.

**Table 4.** Zero-Inflated Poisson Regression Analyses Predicting College Sexual Assault Experiences Between Male and Female Students.

	Male			Female		
	Model 1	Model 2	Model 3	Model 1	Model 2	Model 3
	IRR (95% CI)	IRR (95% CI)	IRR (95% CI)	IRR (95% CI)	IRR (95% CI)	IRR (95% CI)
CCBV	2.73 (1.83, 4.09)***	2.64 (1.62, 4.31)***	3.55 (1.57, 8.06)**	2.35 (2.00, 2.76)***	2.08 (1.74, 2.49)***	1.42 (1.07, 1.88)*
Age	1.15 (0.94, 1.40)	1.24 (1.02, 1.51)*	1.11 (0.98, 1.49)	1.07 (0.96, 1.19)	1.06 (0.95, 1.17)	1.05 (0.95, 1.17)
Race/ethnicity						
Black (non-Hispanic)	0.28 (0.04, 2.25)	4.80 (0.42, 10.38)	3.74 (0.27, 15.67)	0.58 (0.35, 0.94)*	0.75 (0.45, 1.24)	0.78 (0.47, 1.30)
Latinx	3.11 (0.02, 6.01)	1.65 (0.03, 4.36)	1.06 (0.01, 6.93)	0.72 (0.51, 1.01)	0.68 (0.47, 0.97)*	0.70 (0.49, 1.01)
Other	0.26 (0.09, 0.78)*	0.19 (0.04, 0.97)*	0.18 (0.03, 1.15)	0.64 (0.42, 0.98)*	0.63 (0.41, 0.97)*	0.62 (0.41, 0.94)*
Sexual orientation						
Gay/lesbian	3.53 (1.26, 9.89)*	12.93 (3.79, 30.69)***	12.60 (2.32, 29.10)**	0.81 (0.36, 1.83)	0.61 (0.27, 1.39)	0.55 (0.25, 1.24)
Bisexual	1.85 (0.65, 5.23)	5.56 (1.73, 17.83)**	6.89 (1.65, 20.58)**	1.33 (0.89, 1.99)	1.25 (0.83, 1.89)	1.18 (0.78, 1.80)
Prefer not to specify	6.02 (1.60, 12.97)**	4.81 (0.59, 18.97)	6.16 (0.80, 27.12)	0.44 (0.11, 1.77)	0.56 (0.14, 2.28)	0.56 (0.14, 2.30)
Income-based grant	0.65 (0.32, 1.30)	0.72 (0.35, 1.49)	0.70 (0.31, 1.57)	1.60 (1.24, 2.06)***	1.43 (1.11, 1.85)**	1.39 (1.07, 1.81)*
Hours spent online	0.95 (0.86, 1.04)	1.01 (0.92, 1.11)	1.02 (0.93, 1.12)	1.04 (1.01, 1.08)*	1.04 (1.01, 1.08)**	1.05 (1.01, 1.08)**
Online safety	0.87 (0.73, 1.05)	1.02 (0.83, 1.24)	1.03 (0.84, 1.27)	1.01 (0.93, 1.09)	1.00 (0.92, 1.09)	0.99 (0.91, 1.07)
Parental monitoring		0.37 (0.17, 0.79)*	0.24 (0.53, 1.10)		0.60 (0.45, 0.78)***	0.58 (0.38, 0.90)*
Deviant peer association		3.38 (1.73, 6.61)***	5.51 (2.44, 9.63)**		1.31 (0.97, 1.77)	0.77 (0.47, 1.25)
CCBV × parental monitoring			0.41 (0.10, 1.60)			0.41 (0.21, 0.78)**
CCBV × deviant peer association			3.67 (0.18, 7.39)			3.13 (1.71, 5.74)***
χ^2^ (*df*)	88.12 (11)***	118.46 (13)***	121.35 (15)***	134.87 (11)***	155.44 (13)***	172.85 (15)***
Pseudo *R^2^*	0.36	0.48	0.50	0.16	0.18	0.21

*Note.* IRR = incidence rate ratio; CI = confidence interval; CCBV = childhood cyberbullying victimization. Reference groups for race/ethnicity and sexual identity are White and heterosexual/straight, respectively.

*p < .05. **p < .01. ***p < .001.

Model 1 encompassed CCBV, sociodemographic and online use variables. For the total sample, college students who were victims of cyberbullying in childhood reported a higher incidence of sexual assault experiences (IRR = 2.53, *p* < .001). Also, several sociodemographic and online use variables, including age, sex, race/ethnicity, expected family contribution, and hours spent online were associated with incidents of sexual assault experiences in college. Students who were older, female, White, recipients of the income-based grant, and who spent more time online had more experiences of sexual assault. For both male and female students, CCBV increased the incidents of sexual assault experiences (IRR = 2.73, *p* < .001 for males; IRR = 2.35, *p* < .001 for females). Black and other racial/ethnic females, as well as other racial/ethnic males were less likely to report the incidents of sexual assault experiences than White counterparts. Compared to heterosexual sexual orientation male participants, gay male sexual orientation participants and males who preferred not to specify their sexual orientation had higher incidents of sexual assault experiences, while no significant relationship was found for female participants. Female students who were recipients of the income-based grant and spent more time online reported higher incidents of sexual assault experiences, whereas no significant relationship was found for male students.

In Model 2, childhood parent and peer factors were included, and the overall model fits were significant (see Table 3 for total students and Table 4 for males and females). The results showed that CCBV in Model 1 remained significant on sexual assault experiences in Model 2 for the total sample (IRR = 2.20, *p* < .001), males (IRR = 2.64, *p* < .001), and females (IRR = 2.08, *p* < .001), respectively. Parental monitoring significantly decreased the incidents of sexual assault experiences for all three groups, while deviant peer association was a significant predictor to escalate the incidents of sexual assault experiences for the total group and the male group.

Model 3 added two interaction terms, namely CCBV by childhood parental monitoring and deviant peer association (see Tables 3 and 4). Both parental monitoring and deviant peer association were significant moderators that influenced the association between CCBV and college sexual assault experiences for the total group and the female group. Conversely, no significant moderating effect was shown for the male group. More specifically, the positive association between CCBV and sexual assault experiences was significantly weaker among female students with stronger monitoring from their parents than those with less parental monitoring. Similarly, there was a stronger positive association between CCBV and sexual assault experiences among females with higher deviant peer association compared to females with lower deviant peer association (see Figure 1).

**Figure 1.**
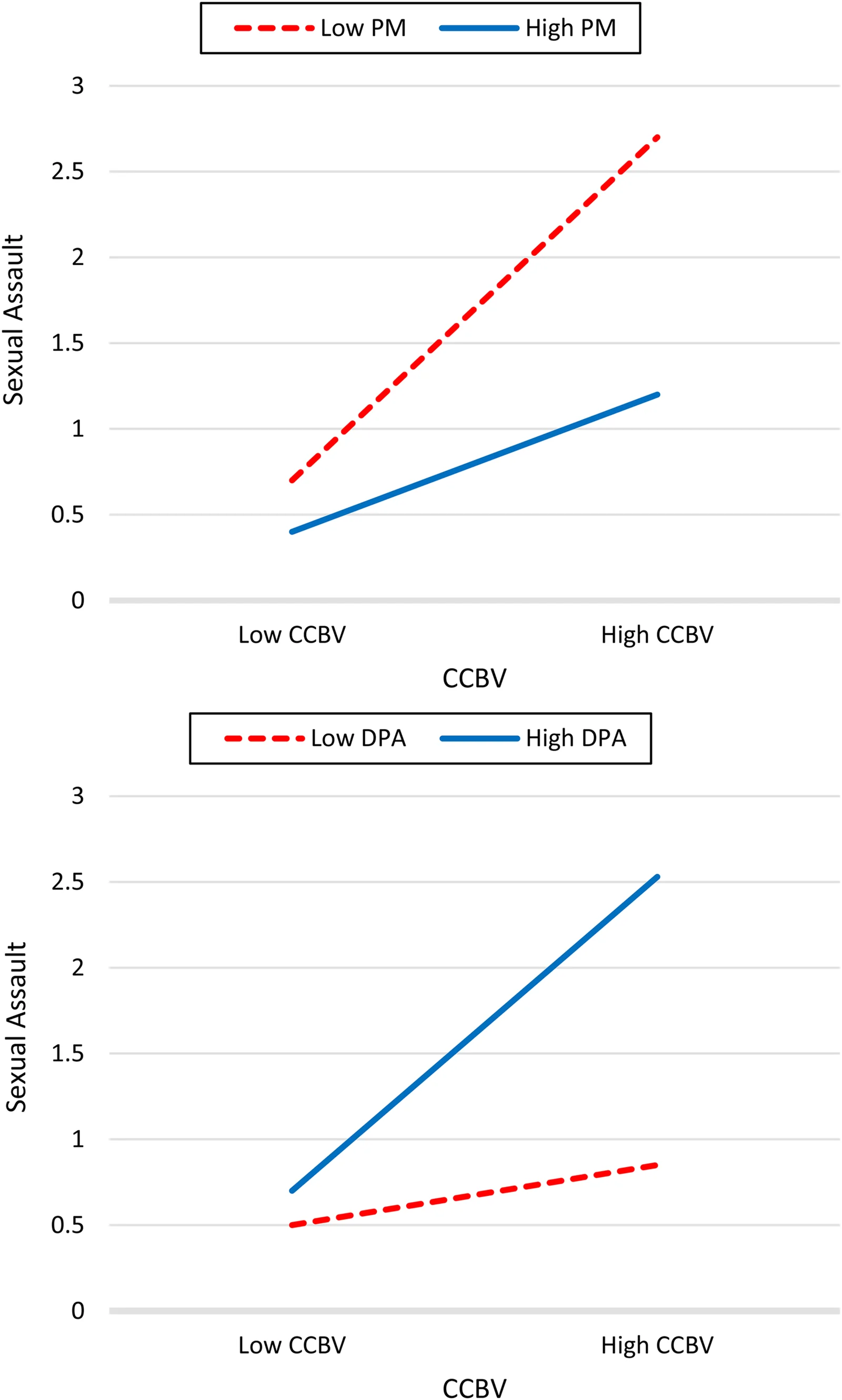
Moderating effects of parental monitoring and delinquent peer association among female students.

*Note.* CCBV = childhood cyberbullying victimization; PM = parental monitoring; DPA = deviant peer association. X-axis ranges from low (−1 SD) to high (+1 SD) levels of CCBV, with simple slopes plotted at low (−1 SD) and high (+1 SD) levels of PM.

## Discussion

This study explored whether the relationship between CCBV and sexual assault experiences differs between males and females in college, whether childhood parental monitoring and peer deviance play moderating roles on the relationship between CCBV and sexual assault experiences, and lastly, how these moderating roles differ between male and female students. The findings from this study found that females were more likely to have sexual assault experiences than males, which supports our research hypothesis and is consistent with previous research (Cantor et al., 2019; Hines et al., 2012). However, our results reported no sex differences in CCBV. Some studies have found sex differences in cyberbullying victimization (Elgar et al., 2014; Kann et al., 2018), whereas others have found no sex differences in cyberbullying victimization (Tokunaga, 2010). With this discrepancy in findings, further studies need to be conducted with data from multiple informants or data with larger sample size.

This study also found that CCBV was positively associated with college sexual assault experiences for both males and females. In line with previous literature on the relationship between CCBV and sexual assault victimization in college (Messman-Moore & Brown, 2004), the findings suggest that both males and female students who were victims of cyberbullying as a child are more likely to experience college sexual assaults. Regarding the covariates, sexual orientation was strongly associated with college sexual assault experiences for male students, and income-based grant and hours spent online were associated with sexual assault experiences for female students. As such, sexual assault experiences may be attributed to not only CCBV, but also sexual orientation for males, and economic hardship and online use for females. Therefore, we should consider cyberbullying preventions in childhood as well as sex-specific programs.

Furthermore, the results indicated that childhood parental monitoring reduced the incidence of sexual assault experiences for both females and males, while childhood deviant peer association increased the occurrence of sexual assault experiences for males only. It is imperative to identify parental monitoring as a salient parent factor that may serve as a protective factor for (alleviate the negative effects of) students’ sexual assault victimization and peer deviance as a critical peer factor that may escalate the risk of (worsen the negative outcomes of) sexual assault victimization. In particular, with higher parental monitoring in childhood comes more parent-child contact, which may facilitate parental support and guidance, as well as provide the young with a secure base to return to, allowing them to be less fearful of standing up for themselves against bullying and sexual violence, regardless of sex (Elsaesser et al., 2017; Jankowski et al., 2002). Meanwhile, deviant peer association is a more worrying issue among males than females. Males who are associated with delinquent peers may tend to be linked with an escalated risk of subsequent sexual victimization because they are readily exposed to violence and abuse in the given climate.

Finally, the findings showed evidence of both childhood parental monitoring and deviant peer association playing moderating roles on the relationship between CCBV and sexual assault experiences for the female group, but not for the male group. Interestingly, parental monitoring was a significant moderator to attenuate the positive association between CCBV and sexual assault experiences, whereas deviant peer association was a moderator that strengthened the association between CCBV and sexual assault experiences among female students. These results are consistent with prior research which confirmed the moderating roles of either parental monitoring or deviant peer relationship (Hood & Duffy, 2018; Lee et al., 2018; Wright, 2016). Given that parental monitoring and deviant peer association were significant moderators for the female group, we can conclude that parent and peer factors are valuable in weakening or reinforcing the association with CCBV and sexual assault experiences among females. In fact, parent and peer factors have been proven to be effective in understanding cyberbullying victimization and sexual assault victimization in other research as well (Franklin et al., 2012; Loh & Gidycz, 2006). Thus, a positive parent-child relationship and peer support may contribute to reducing cyberbullying victimization and sexual assault experiences, especially among female students.

### Limitations

Although our findings expand existing knowledge on the relationship between CCBV and sexual assault experiences, there are some limitations in our study that warrant caution. First, with the nature of the cross-sectional data, the causal effects of CCBV on college sexual assault experiences were precluded in this study. Further research using longitudinal dataset should be considered to evaluate the long-term effects of CCBV on sexual assault experiences as well as the moderation effects of parental monitoring and deviant peer relationship. Second, self-report measures utilized in this study may present social desirability bias and method variance effects. In addition, several childhood measures (e.g., CCBV, childhood parental monitoring) were retrospective, depending on the recall of personal memories, which increases the risk of response bias by distorting childhood memories. Future studies should be considered using multiple sources of measures, such as parent and teacher reports, to reduce the recall bias. Third, the oversample of female participants in the current study may be construed as a skewed sample.

Fourth, the sample included only juniors and seniors, which limits the generalizability of the findings to freshmen and sophomores, who may experience difference timing and patterns of sexual assault risk. Future research should include freshmen and sophomores to examine whether the associations between childhood cyberbullying victimization and sexual assault differ by year in school. Fifth, our measure of childhood parental monitoring warrants careful interpretation, as the items referred broadly to parents without distinguishing the types of caregivers, such as biological parents, grandparents, foster carers. Thus, it would be well understood as perceived monitoring by a primary caregiver and may function as a proxy for broader caregiver involvement rather than a specific parent role. Moreover, scores were relatively high and clustered at the upper end of the scale (M = 4.45 on a scale of 5), suggesting possible ceiling effects and restricted variability that may have attenuated the association and moderating effects observed in this study. Sixth, the current study could not control for historical context in relation to the time point when participants first had access to online social spaces and/or experiences cybervictimization. Seventh, the measure of college sexual assault relied on an adapted SES with behaviorally specific item but did not provide detailed definitions for terms such as “sex play,” “intercourse,” and “rape,” and did not include all possible forms of sexual victimization. As such, it may not fully capture all victims and likely underestimates the prevalence and diversity of sexual assault experiences in this sample. Future study should consider using more comprehensive campus climate-related instruments. The last notable limitation of this study is the exclusive focus on CCBV without accounting for other types of childhood adversity or victimization. While CCBV emerged as a significant predictor in the model, it may be part of a broader constellation of adverse childhood experiences (ACEs). The absence of data on other ACEs, such as physical and emotional abuse, neglect, or exposure to domestic violence, limits the ability to fully understand the unique and cumulative effects of CCBV. Future studies should adopt a more comprehensive approach by adding multiple dimensions of childhood adversity to examine the extent to which CCBV functions as a distinct or compounding ACE.

### Implications for Practice

Childhood parental monitoring appears to play an important role in reducing subsequent sexual assault experiences among female college students who experienced cyberbullying in their childhood. Interventions should therefore focus on enhancing consistent, supportive monitoring and communication within whatever caregiving context students are embedded. For example, research has found that communication during the college years between mothers and daughters was correlated with lower rates of sexual victimization (Testa et al., 2010). It is possible that parents who engage in parental monitoring also engage in other types of communication that may have preventative effects. Therefore, child-parent communication patterns over a longer period should be explored to inform parental interventions.

The current study suggests that parents may play an important role in mitigating the risk of sexual assault. Thus, social service practitioners and educators should include parents in interventions aimed at mitigating the effects of cyberbullying. For instance, parents can help children identify the seriousness and emotional impacts of cyberbullying on the victims. Harper (2019) found that cyberbullying has become normative among adolescents because they may view it simply as “drama”, making it difficult for them to recognize it as bullying. Parents, on the other hand, are more likely to identify cyberbullying as a form of bullying and understand its implications on their children's wellbeing. As such, ongoing parent-child communication is essential in helping a child identify cyberbullying and assisting him/her in addressing it.

Social service practitioners can guide parents on how to impart knowledge regarding emotional regulation to their children, so that their children are able to appropriately address and manage the emotions that arise due to cyberbullying (Nijenhuis et al., 2004). Addressing the trauma resulting from cyberbullying is essential because young people with untreated trauma have difficulty identifying risk and they often feel powerless, which are related to responding to future threats of sexual assault with non-assertive behaviors (e.g., freezing, submission) (Gidycz et al., 2008).

Beyond the family context, the findings have broader policy implications for schools, universities, and community systems. As childhood cyberbullying victimization is linked to later sexual assault experiences, policies that integrate cyberbullying prevention with sexual violence prevention across developmental stages are warranted. In K–12 settings, this includes incorporating digital citizenship, cyberbullying awareness, and trauma-informed responses into school discipline and student support policies. At the college level, campus sexual assault policies and prevention programs may benefit from explicitly recognizing prior cyberbullying victimization and other adverse childhood experiences as risk markers, and from ensuring that counselling, advocacy, and reporting systems are equipped to highlight overlapping online and offline victimization. Partnerships between universities, community agencies, and caregiver systems can help create coordinated pathways of support for students who have experienced cyberbullying and sexual assault.

## Conclusion

In summary, cyberbullying is an emerging psychosocial issue for young people because of its ability to follow children beyond school walls and it causes negative consequences parallel with those of traditional bullying. As cyberbullying has been found to be a major predictor of psychological and emotional stresses later in adulthood, it is important that family support remains intact as a support system, especially when peer support is negligible. Moreover, childhood parental monitoring is a critical element that can reduce subsequent sexual assault among female college students who had experienced cyberbullying in their youth. Ultimately, childhood bullying is a traumatic experience that can significantly impact the future of victims unless otherwise combatted by peer support and parental monitoring.
